# Magnitude and Associated Factors of Awareness with Recall under General Anesthesia in Amhara Regional State Referral Hospitals, 2018

**DOI:** 10.1155/2019/7043279

**Published:** 2019-07-07

**Authors:** Tadese Tamire, Habtamu Demelash, Tikuneh Yetneberk, Simegnew Kibret

**Affiliations:** ^1^College of Health Sciences, Department of Anaesthesia, Debre Tabor University, Debra Tabor, Ethiopia; ^2^College of Health Sciences, Department of Public Health, Debre Tabor University, Debra Tabor, Ethiopia

## Abstract

**Introduction:**

Awareness with recall of intraoperative events is an infrequent but potentially devastating complication of general anesthesia, with a reported incidence of 0.1-0.2% in low-risk patients. Higher incidence is expected in resource-limited operation room setups and in high-risk patients. Awareness can result in significant distress to patients and long-term psychological consequences, including symptoms associated with posttraumatic stress disorder, anxiety, night mares, night terror, dissatisfaction with surgical service, and sometimes even suicide.

**Objective:**

To assess the magnitude and associated factors of awareness with recall under general anesthesia in Amhara regional state referral hospitals.

**Method:**

An institution-based cross-sectional study was conducted on 1065 patients who underwent surgery under general anesthesia from January 1 to June 30, 2018. The study participants were selected by systematic random sampling from 4 referral hospitals. The modified Brice questionnaire was used to detect awareness under general anesthesia. Interviewer-administered structured questionnaire and chart review were employed. Data with complete information were entered in to SPSS version 20 computer software. Descriptive statics and bivariate and multivariable analysis were computed. A *P*-value less than 0.2 was used to select candidate variables for multivariable logistic regression. A *P*-value less than 0.05 was used to declare statistical significance.

**Result:**

1065 patients were included in the study which makes the response rate of 90.7%. The magnitude of awareness with recall under general anesthesia was found to be 8.2% of which 4.9%, 2.6%, and 0.7% of patients reported hearing voice, pain, and sensation of breathing tube, respectively. Lack of premedication was the only significantly associated factor for awareness with recall under general anesthesia (AOR = 3.014, 95% CI (1.201 to 7.565)).

**Conclusion and Recommendation:**

Our study showed higher magnitude of awareness with recall under general anesthesia. Lack of premedication was the only associated factor with awareness with recall under general anesthesia. Anesthetists should give emphasis to prevent the possibility of awareness under general anesthesia by providing premedication. Cohort studies should be done including the consequences of awareness with recall under general anesthesia.

## 1. Introduction

General anesthesia is an induction of a state of unconsciousness with the absence of sensations over the entire body through the administration of anesthetic drugs for certain medical and surgical procedures. The purposes of general anesthesia include pain relief, blocking memory of the procedure (amnesia), producing unconsciousness, inhibiting normal body reflexes to make surgery safe, and easier to perform by relaxing the muscles of the body [1].

Anesthesia awareness, also called intraoperative awareness, is defined as unintended explicit recall of intraoperative events [2]. Administration of general anesthesia started in the nineteenth century for the first time in public demonstration of William Morton of the Massachusetts General Hospital for the procedure of tooth extraction for Gilbert Abbot. After the procedure, Abbot said that he remembered but did not feel pain [3]. From these, it can be noted that the problem exists starting from the time of first administration of the first anesthetics. Awareness under anesthesia with postoperative recall most of the times declared by patients as their worst medical experience can lead to long-term psychological injury such as anxiety, insomnia, nightmare, irritability, and depression possibly leading to suicide [4].

Generally, the possible traumatic psychological sequalae of this complication were not acknowledged in the medical literature until the 1970s [2]. However, despite several decades of attention to this problem, patient groups and, especially, individual patients at risk have not been clearly identified, the definition of this complication is not entirely clear, its prevention has not been studied in detail, and the nature and incidence of possible after-effects of awareness during general anesthesia are not known [3]. Especially in our country where there are no modern anesthetic drugs, no modern monitoring modalities to know the depth of anesthesia, and very few trained anesthetists who can recognize this complication, there is no information on the magnitude of awareness with recall under general anesthesia in Ethiopia. In low-income countries including Ethiopia, the commonly used anesthetic drugs to provide general anesthesia include: atropine, diazepam, ketamine, thiopentone, propofol, and halothane. Even though the above drugs can prevent the possibility of awareness with recall during general anesthesia, the attention given in preventing awareness is very limited in our observations. This is may be because of lack of evidence on the incidence of the problem. The routinely used intraoperative monitoring devices in low-income countries include noninvasive blood pressure monitor and pulse-oximetry. ECG and capnograph are used sometimes. None of the above monitoring devises can measure the level of consciousness under general anesthesia. Moreover, the majority of anesthesia service is being provided by nonphysician anesthetists after short duration of anesthesia trainings.

Thus, this multicenter cross-sectional study examined the magnitude and risk factors of awareness with recall under general anesthesia among surgical patients in Amhara regional state referral hospitals from January 1 to June 30, 2018.

## 2. Methods

An institution-based cross-sectional study was conducted in 4 Amhara regional state referral hospitals, North of Ethiopia. Amhara regional state has 5 referral hospitals which are providing services for a population of around 25 million. These hospitals provide services like diagnosis, treatment, and referral in different specialties for about 300,000 to 400,000 patients per year. A total of 20,000 to 25,000 patients are estimated to undergo surgery in a year. This study was conducted from January 1 to June 30, 2018. All adult postoperative patients who underwent surgery under general anesthesia were included in this study. Critically ill patients or patients admitted to ICU were excluded from this study.

### 2.1. Sample Size Determination

The sample size was determined using a single population proportion formula by considering 50% incidence of awareness under general anesthesia (because we could not find any literature on the prevalence of awareness with recall under general anesthesia in our country Ethiopia) at 95% confidence interval and 3% margin of error. We decreased the margin of error to increase the sample size which enables us to detect this rare problem as reported from data from abroad. Then, 10% of nonresponse rate was added on calculated sample size. The total sample size was determined to be **1174** patients.

### 2.2. Sampling Technique

A systematic random sampling technique was used in each hospital until the predetermined sample size for the hospital is achieved. An average surgical patient flow for each referral hospital was determined by reviewing the anesthesia and surgical logbooks. Then, the average monthly patient flow was calculated based on the 6-month report. The number of study participants were allocated proportionally based on their patient flow. Then, the skipping interval was calculated to select participants from each hospital. The skipping interval was calculated for each hospital based on the patient flow until the predetermined sample size is achieved.

### 2.3. Variables

The dependent variable of this study was awareness with recall under general anesthesia. The independent variables includes sex, age, ASA physical status, drugs given as a premedication, induction agents, neuromuscular blockers, use of opioids, urgency of surgery, and the category of surgical procedure.

### 2.4. Data Collection Technique

A semistructured questionnaire was prepared to collect data on demographic characteristics, patient-related variables, anesthesia-related variables, and surgery-related variables after referring to different studies. A modified Brice questionnaire was used to detect awareness with recall under general anesthesia. Participants were interviewed within 2 to 24 hours after they woken from general anesthesia which was confirmed by the Aldrete score. An interviewer-administered questionnaire and their chart including anesthetic record sheet which was reviewed was used. The data collectors were selected from Bachelor of Science in clinical nursing who have experience of working in postanesthesia care unit and surgical wards. Two data collectors and one supervisor were allocated for each hospital.

### 2.5. Data Quality Assurance

To ensure the quality of data, one-day training was given to the data collectors and supervisors. A pretest study was conducted on 5% of the sample size at the Debre Tabor General Hospital to assess the data collection instrument and challenges of data collection process. Then, we reviewed our data collection tool for the main research data collection. The questionnaire was translated to Amharic, and then trained data collectors collected the data carefully under close supervision of the supervisors. Collected data were checked for completeness, clarity, consistency, and accuracy by the supervisors and the principal investigators.

### 2.6. Data Analysis

Data with complete information were entered into SPSS version 20 and cleaned. Descriptive statistics and association between the independent factors and the outcome variable were computed at 95% confidence interval.

Both bivariate and multivariable logistic regressions were employed to assess the association between the outcome and explanatory variables. Crude and Adjusted Odds Ratio were used to see the strength of the association. A *p*-value of less than 0.05 was used to declare statistical significance.

### 2.7. Ethical Considerations

Before conducting the study, ethical approval was obtained from the Debre Tabor University ethical review committee and permission was obtained from each hospital's administrators after providing detailed information about the objectives of our study. After getting permission from hospital managers, data collectors took written informed consent from each patient to collect the data. Participants were informed that they have the right to withdraw from the research process if they feel discomfort before they provide any information. During the data collection process, norms, values, and morals of patients were respected by data collectors.

## 3. Results and Discussion

### 3.1. Demographic Characteristics of the Study Participants

A total of 1065 patients with ASA I to III participated from 4 referral hospitals with the response rate of 90.7%. 53% of the participants were male, and the mean age of the study population was 39.39 with SD of 18.8. Ninety-one % of patients were between the ages of 18 to 65 years; whereas the remaining 9% were above 65. Fiftey three % of surgeries were done as an elective surgery. The distribution of surgical procedures performed during the study period is shown in the Table 1.

### 3.2. Magnitude of Awareness with Recall under General Anesthesia

In this study, the magnitude of awareness with recall under general anesthesia was found to be 8.2%. Ketamine was the frequently used induction agent followed by propofol (42.7% and 25.6%, respectively). Tramadol, pethidine, and diclofenac were the drugs used most frequently as premedication and analgesics. Halothane was the widely used (85.7%) inhalational agent for maintenance of general anesthesia. 81.7% of patients were given muscle relaxants both for intubation and for the procedure. Eighty-one (7.6%) patients were reported dreaming under anesthesia. But, there was no association between dreaming and the incidence of awareness with recall under general anesthesia. In this study, dreaming is not considered as awareness with recall under general anesthesia. Patients who reported dreaming were asked whether their dream was disturbing them, but none of them were reported that their dream is disturbing them. In the following table (Table 2), we assessed the intraoperative events that can be considered as a positive response for awareness under general anesthesia. Pain and anxiety were the worst things reported by patients in their operation (27.3% and 26.5%, respectively).

Most patients who reported awareness with recall under general anesthesia were reported hearing voices during surgery followed by feeling pain.

### 3.3. Associated Factors

We computed binary logistic regression to assess the strength of associations between the dependent variable and independent variables (Table 3) to determine factors associated with awareness with recall under general anesthesia.

The multivariable logistic regression analysis showed (Table 3) that patients who are not premedicated with benzodiazepines or atropine were at increased risk of awareness with recall under general anesthesia (AOR of 3.014 (CI: 1.201 to 7.565)).

None of the other factors showed significant association for awareness with recall under general anesthesia. This may be because of the uneven distribution of patients and small sample size.

## 4. Discussion

Typical positive responses indicating awareness are factual memories of discussions, noises, tactile sensations, and feeling of muscle paralysis or episodes of pain or distress that can be traced back to the intraoperative period [5]. In the current study, we defined awareness with recall under general anesthesia if a patient recalls two or more of the above positive responses. Based on this definition, our study revealed 8.2% of awareness with recall under general anesthesia, of which 4.9% were reported hearing voices, whereas 2.6% were reported feeling pain (Table 2). Generally, this study showed high magnitude of awareness with recall under general anesthesia which calls for attention of anesthetists in preventing or at least minimizing this problem.

The finding is comparable with some older studies conducted on high risk patients like cardiac surgery, cesarean section, endoscopic procedures, and trauma surgeries [6–8]. Barr and Wong [9] reported an incidence of awareness and recall of 4.0% in patients anesthetized with thiopental for broncho- or laryngoscope [10]. In another study by Moore and Seymour (1987) that investigated awareness during bronchoscope, the incidence of awareness with recall was 6.7% in a group of 104 patients [7].

On the other hand, our study showed by far higher magnitude of awareness with recall under general anesthesia compared to other recent studies in USA and European countries [10–14]. In 1973, McKenna and Wilton tried to find out the incidence of awareness and recall during intubation. 160 patients were interviewed; three (1.9%) reported intraoperative awareness [15]. However, only two were able to remember the intubation while the third patient recalled paralysis. In another study by Pierre [13], 30 patients were anesthetized with different doses of etomidate (0.2–0.4 mg/kg) and fentanyl [16]. The patients' consciousness was monitored during intubation by the isolated forearm technique (IFT). Positive IFT response was found in 80, 70, and 20% of the patients receiving 0.2, 0.3, and 0.4 mg/kg etomidate, respectively.

One of the patients with positive IFT response recalled awareness in a postoperative interview, giving an incidence of awareness with recall of 3.3% during intubation [3, 15]. In our study, we could not estimate the dose of anesthetic agents used because the weight of patients was not recorded or measured.

In the current study, 81.7% of patients were given muscle relaxant, about 47% of patients were operated as an emergency surgery, and 84% of patients were not given amnesic drugs like benzodiazepines and anticholinergics. Benzodiazepines and anticholinergics like scopolamine and atropine have amnesic effects which inhibit the recall of awareness under general anesthesia. Therefore, the higher magnitude of awareness in our study may be due to the anesthetic technique used, dose of anesthetic agents, and the type and time of operation [16, 17].

Furthermore, the timing of postoperative interview to detect awareness is also a matter of debate on the magnitude of awareness with recall under general anesthesia. Some studies like Brice et al.'s [10] and others showed that early interviewing increases the incidence of awareness with recall under GA, whereas Errando et al. and his colleagues [18] showed decreased incidence of awareness with recall under GA when interviewed later [9, 14, 19, 20]. On the other hand, Sandin and coworkers suggested that interviewing more than once will increase the number of detected cases of awareness [6]. In the current study, we interviewed patients only once within 2 to 24 hours of postoperative period when the patients are fully awake from general anesthesia which was determined by the Aldrete score. We believe that if patients were interviewed again few days after the first interview, it could have been used to confirm the occurrence of awareness with recall under general anesthesia. But, we could not do this because of the financial constraints. So, we would like to recommend other researchers to interview patients more than once for better diagnosis of awareness with recall under general anesthesia. In the current study, patients reported pain and anxiety as the worst experiences they had in their operation (27.3% and 26.5%, respectively). This is may be due to lack of strong opioids in the study areas and poor preoperative patient assessment and assurance in alleviating pain and anxiety of patients.

Regarding the factors associated with awareness under general anesthesia, our study showed that lack of premedication was the only significantly associated factor for awareness with recall under general anesthesia. This finding is not in line with other studies in which higher incidence and/or associations were reported in the following patient groups: use of alcohol, substance abuse, light anesthesia, higher ASA status, trauma surgery, cardiac surgery, C/S, emergency surgery, and endoscopies [11, 17, 21–24]. This may be because of the uneven distribution of the patient characteristics and small sample size in our study patients.

### 4.1. Limitations of the Study

In this study, detailed interview of patients who reported awareness with recall under general anesthesia was not done which could help us to confirm detection of awareness with recall under anesthesia. The other limitation of this study is that the interview was not repeated; repeating the interview may also support the diagnosis. Even though it is not our objective in the current study, it will be more powerful if we had assessed the consequences of awareness with recall under general anesthesia.

## 5. Conclusion

The current study revealed a very high magnitude of awareness with recall under general anesthesia compared to other studies. Hearing voice and pain were the two common intraoperative events recalled by patients who had awareness with recall under general anesthesia. From all patients involved in this study, pain and anxiety were the worst experiences reported by patients during their operation (27.3% and 26.5% respectively). Lack of premedication with benzodiazepines or atropine was the only significantly associated factor for awareness with recall under general anesthesia (AOR = 3.014, 95% CI (1.201 to 7.565).

## 6. Recommendation

Anesthetists should give great emphasis to prevent the possibility of awareness with recall under general anesthesia and should premedicate patients with amnesic drugs. Emphasis should also be given to treat pain and alleviate the anxiety of patients. Researchers should conduct cohort studies including the consequences of awareness with recall under general anesthesia.

## Figures and Tables

**Table 1:** Frequency and percentage of surgical procedures performed in the study participants in 4 Amhara regional state referral hospitals from January 1 to June 30, 2018.

Procedures	Frequency	Percent
Neurosurgery	30	2.8
Cardiothoracic	10	0.9
ENT surgery	22	2.1
Thyroidectomy	128	12.0
Abdominal surgeries	581	54.6
Cesarean section	94	8.8
Trauma	87	8.2
Orthopedics	73	6.9
Others	37	3.5
Total	1065	100.0

The majority of surgical procedures done during the study period were abdominal surgery followed by thyroidectomy (54% and 12%, respectively).

**Table 2:** Frequency and percentage of intraoperative events reported among patients who had awareness with recall under general anesthesia in 4 Amhara regional state referral hospitals from January 1 to June 30, 2018.

Events	Frequency	Percentages
Hearing voices	52	4.9
Sensing surgical events	9	0.8
Unable to move or breath	12	1.1
Anxiety or stress	19	1.8
Feeling pain	28	2.6
Sensation of breathing tube	7	0.7
Others	2	0.2
Total	1065	100.0

**Table 3:** Multivariable logistic regression among patients who develop awareness with recall under general anesthesia in Amhara regional state referral hospitals, 2018.

	Sig.	Exp(B)	95% C.I. for EXP(B)	
			Lower	Upper
Age of patient	0.725	1.155	0.517	2.580
Sex of patient	0.833	1.053	0.649	1.710
ASA status	0.092	1.510	0.935	2.437
History of alcohol	0.835	1.01	1.45	3.02
History of chronic pain	0.904	1.050	0.471	2.340
Premedication	0.002	3.014	1.201	7.565
Difficult intubation	0.982	0.991	0.458	2.146
Use of inhalational drugs	0.358	0.555	0.158	1.947
Use of muscle relaxant	0.593	1.207	0.605	2.410

## Supplementary Materials

Supplementary Materials Description of the Questionnaire: the following questionnaire is prepared after reviewing different studies on awareness with recall under general anesthesia. We used the modified Brice questionnaire which was developed in 2014 to assess awareness with recall under general anesthesia. So, we provided the general information given below to help each participant in making informed decision to participate on this research. The questionnaire has three parts which include patient identification, Brice questionnaire to detect awareness with recall under general anesthesia, and risk assessment.
