# Supplementary material for: Magnitude and Associated Factors of Awareness with Recall under General Anesthesia in Amhara Regional State Referral Hospitals, 2018
Source: Anesthesiol Res Pract. 2019 Jul 7;2019:7043279. doi: 10.1155/2019/7043279 (PMC6642758; doi:10.1155/2019/7043279)
Supplement: Supplementary Materials — Description of the Questionnaire: the following questionnaire is prepared after reviewing different studies on awareness with recall under general anesthesia. We used the modified Brice questionnaire which was developed in 2014 to assess awareness with recall under general anesthesia. So, we provided the general information given below to help each participant in making informed decision to participate on this research. The questionnaire has three parts which include patient identification, Brice questionnaire to detect awareness with recall under general anesthesia, and risk assessment. [file 7043279.f1.pdf]

Indexes

I. Questionnaire

Debre Tabor University

College of Health Sciences

Department of Anesthesia

Magnitude and Associated Factors of Awareness with Recall under General Anesthesia in Amhara Regional State Referral Hospitals

<u>General Information</u>

My Name is_____________; I'm here on behalf of Debre Tabor University. The aim of this questionnaire is to assess the magnitude and associated risk factors of awareness under general anesthesia among surgical patients in Amhara regional state referral hospitals. This study will help to fill the information gap on the magnitude and risk factors of awareness under general anesthesia which can contribute for the improvement of Perioperative anesthesia management and increase patient satisfaction on anesthesia service. You are randomly selected to participate on this study and provide appropriate response to questions. Your participation is voluntary. Only anonymous data will be analyzed and we strictly keep confidentiality of participants. Participating or not participating on this study will not bring any harm or benefit to you. This interview will take a maximum of 15 minutes. We strongly value your honest response for the questions. If you feel or face any problem regarding your participation, you can contact the principal investigators by 0913724684. Do you agree to participate?

1. If agree go to the next page
2. If not agree got to the next participant

Name of the data collector: ____________________ Date: ___________ Sig. ________

Name of the supervisor: _______________________ Date: ___________ Sig. ________

Part I: Identification			
**S. No**	**Questionnaire**	**Response**	**Remark**
1001	Code No	/____________/	
1002	Age	/____________/year	
1003	Sex	1. Male 2. Female	
1004	ASA status	1. I 2. II 3. III 4. IV 5. V	
**Part II: Modified Brice Questionnaire, 2014**			
2001	Were you expecting to be completely asleep for this operation?	1. Yes 2. No	
2002	What is the last thing you remember before going to sleep?	1. Being in the pre-op area<br>2. Seeing the operating room<br>3. Being with family<br>4. Hearing voices<br>5. Feeling mask on face<br>6. Smell of gas<br>7. Burning or stinging in the IV line<br>8. Other /________________/	
2003	What is the first thing you remember after waking up?	1. Hearing voices<br>2. Feeling breathing tube<br>3. Feeling mask on face<br>4. Feeling pain<br>5. Seeing the operating room<br>6. Being in the recovery room<br>7. Being with family<br>8. Being in ICU<br>9. Nothing<br>10. Other /_______________/	
2004	What do you remember between going to sleep and waking up?	1. Nothing<br>2. Hearing voices<br>3. Hearing events of the surgery<br>4. Unable to move or breathe<br>5. Anxiety/stress<br>6. Feeling pain<br>7. Sensation of breathing tube<br>8. Feeling surgery without pain<br>9. Other /_______________/	
2005	Did You dream during your procedure?	1. No 2. Yes	
2006	If yes, what was your dream?	/__________________________<br>__________________________<br>__________________________/	

2007	Were your dreams disturbing to you?	1. No 2. Yes	
2008	What was the worst thing about your operation?	1. Anxiety<br>2. Pain<br>3. Recovery process<br>4. Unable to carry out usual activities<br>5. Awareness<br>6. Other /_______________/	
**Part III: Assessment of Risk Factors**			
3001	Do you drink alcohol?	1. Yes 2. No	
3002	If your answer for Q3001 is yes, how often do you drink?	1. Rarely<br>2. Sometimes<br>3. Usually<br>4. Always	
3003	Do you have history of chronic pain?	1. Yes 2. No	
3004	If your answer for 3003 is yes, are you taking medication for your pain?	1. Yes 2. No	
3005	Do you have previous history of awareness with recall under anesthesia?	1. Yes 2. No	
**To be filled from the anesthetic record**			
3006	Was the patient intubated?	1. Yes 2. No	
3007	Was the intubation RSI (rapid sequence induction)?	1. Yes 2. No	
3008	Was the patient premedicated?	1. Yes 2. No	
3009	If the answer for Q3 is Yes, what were the medication used and its dose?	/--<br>----------------------/	
3010	Was the intubation difficult?	1. Yes 2. No	
3011	Was inhalational drug(s) used?	1. Yes 2. No	
3012	If yes, what is the name of the inhalational drug used?	/________________________/	
3013	Had the patient had given muscle relaxant for intubation?	1. Yes 2. No	
3014	Had the patient given muscle relaxant for maintenance/during the procedure?	1. Yes 2. No	

3015	Had the patient given opioids (s)?	1. Yes 2. No	
3016	Other anesthetic drugs used and their dose: (from anesthetic record)	a) For induction -----------<br>b) For analgesia--------------<br>c) For muscle relaxation--------<br>d) For maintenance-------------	
3017	Type of surgery	1. Emergency 2. Elective	
3018	What was the category of surgical procedure done?	1. Neurosurgery<br>2. Cardiothoracic surgery<br>3. ENT surgery<br>4. Thyroidectomy<br>5. Abdominal surgery<br>6. Caesarian section<br>7. Trauma surgery<br>8. Orthopedic surgery<br>9. Other (specify)/___________/	

III. Declaration

We, the under signed investigators declare that this research is their original work.

Name:

1. ________________________________ Signature: _____________________
2. ________________________________ Signature: _____________________
3. ________________________________ Signature: _____________________
4. ________________________________ Signature: _____________________

Place of submission: Office of Research and community Service Coordinator of College of Health Sciences, Debre Tabor University.

Date of Submission: ___________________________________
